# Sex-specific cardiometabolic risk markers of left ventricular mass in physically active young adults: the CHIEF heart study

**DOI:** 10.1038/s41598-022-15818-y

**Published:** 2022-07-07

**Authors:** Kun-Zhe Tsai, Pang-Yen Liu, Wei-Chun Huang, Joao A. C. Lima, Carl J. Lavie, Gen-Min Lin

**Affiliations:** 1 Department of Internal Medicine, Hualien Armed Forces General Hospital, No. 100, Jinfeng St., Hualien City, 970 Taiwan; 2 Department of Stomatology of Periodontology, Mackay Memorial Hospital, Taipei, Taiwan; 3 Department of Medicine, Tri-Service General Hospital, National Defense Medical Center, Taipei, Taiwan; 4 College of Medicine, National Yang Ming Chiao Tung University, Taipei, Taiwan; 5 Department of Critical Care Medicine, Kaohsiung Veterans General Hospital, Kaohsiung, Taiwan; 6 Departments of Cardiology and Radiology, Johns Hopkins University School of Medicine, Baltimore, MD USA; 7 Ochsner Clinical School, John Ochsner Heart and Vascular Institute, The University of Queensland School of Medicine, New Orleans, LA USA

**Keywords:** Cardiology, Risk factors

## Abstract

Greater physical fitness may lead to greater left ventricular mass (LVM) and reduce the effect of cardiometabolic risk factors on LVM. However, the cardiometabolic biomarkers associations for LVM have not been clarified in physically active young adults. This study included 2019 men and 253 women, aged 18–43 years, from the military in Taiwan. All participants underwent anthropometric and blood metabolic markers measurements, and completed a 3000-m run test for assessing fitness. LVM was calculated on the basis of an echocardiography. Multiple linear regression was used to determine the sex-specific associations between cardiometabolic risk markers and LVM indexed for the body height (g/m^2.7^). In men**,** age, systolic blood pressure (SBP), 3000-m running time, serum triglycerides, serum uric acid and waist circumference (WC) were correlated with LVM index (β = 0.07, 0.10, − 0.01, 0.01, 0.24 and 0.24, respectively; all *p*-values < 0.05). The correlations were not significant for fasting plasma glucose, total cholesterol and high-density lipoprotein cholesterol (HDL-C). In women, SBP, HDL-C and WC were correlated with LVM index in the univariate analysis (β = 0.07, − 0.05 and 0.32, respectively; all *p*-values < 0.05), whereas the correlation was only significant for WC in the multiple linear regression analysis (β = 0.20; *p*-value < 0.001). In physically active adults, the associations of cardiometabolic risk markers with LVM might vary by sex. Better endurance exercise performance associated with greater LVM was noted only in men, while greater WC was the only metabolic risk marker for greater LVM in both men and women.

## Introduction

Cardiometabolic risk markers are a collection of abnormalities characterized by abdominal obesity, hypertension, dyslipidemia, hyperglycemia and increased oxidative stress^1^. The risk markers may contribute to end-organ damage, such as left ventricular hypertrophy (LVH)^2,3^. Cigarette smoking, alcohol intake, low physical fitness and cardiometabolic abnormalities have been considered as the modifiable risk factors of CVD^4–8^. LVH may act as a role of mediator to CVD^9^, and can be a sign of patients with increased risk of CVD where physicians may take a more aggressive action on CVD prevention. Prior studies have revealed that the contributions of the cardiometabolic risk markers to LVH or CVD may vary by age and sex^10,11^. For instance, serum triglycerides have been associated with left ventricular mass (LVM) in hypertensive individuals, which might be only observed in men rather than women^11^. Therefore, it is critical to investigate the associations between the cardiometabolic risk markers and LVH in specific populations.

As is known, endurance and muscular strength exercises can modulate cardiac structure, resulting in greater LVM, and improve cardiac diastolic function^12^. The degree of cardiac remodeling related to exercises may vary by race and sex^13–15^. Prior studies have uncovered that for a given level of physical training, African/Afro-Caribbean athletes show more prominent LVM changes than do Asian and Caucasian athletes^12–15^, due in part to genetic variations. In addition, male athletes have been observed with a more marked increase in LVM after training compared to female athletes^13,16^. The effect and interplay between physical fitness and the risk factors of CVD on LVM changes have been rarely examined in physically active individuals, such as athletes and military recruits. We hypothesized that physical fitness could reduce the association between the cardiometabolic risk markers and LVM assessed by electrocardiography (ECG) and echocardiography. The purpose of the study was to clarify the associations in military men and women in Taiwan.

## Methods

### Study population

The cardiorespiratory fitness and health in eastern armed forces (CHIEF)-heart study included 2688 military personnel, aged 18–43 years, in whom 2386 were men and 302 were women in Eastern Taiwan of R.O.C., 2018–2021^17–19^. All male and female participants were physically active, and received daily aerobic and resistance exercise training. All subjects underwent annual military health examinations, including laboratory tests and a self-reported questionnaire for their behavior of toxic substances use, i.e., cigarette smoking and alcohol intake (former or never vs. active) in the Hualien Armed Forces General Hospital. All subjects participated annual fitness exams in the Military Physical Training and Testing Center in Hualien for examining endurance capacity by the time for a 3000-m run test. All participants received a 12-lead ECG and transthoracic echocardiography for evaluating cardiac structure following the exercise test and before the end of index year^19,20^. Participants who had serum triglycerides ≥ 400 mg/dL (N = 38), blood pressure (BP) ≥ 140/90 mmHg (N = 346), or body mass index (BMI) ≥ 35 kg/m^2^ (N = 32) were excluded, leaving a sample of 2019 men and 253 women for analysis.

### Anthropometric, hemodynamic and blood test measurements

Body height, body weight and WC measurements of all study subjects were carried out in a standing position. BMI was calculated as body weight (kg) divided by square of body height (m^2^). The blood pressure (BP) of each subject at rest was measured over right arm in a sitting position by the automatic BP monitor machine (FT201 Parama-Tech Co., Ltd, Fukuoka, Japan), which used the oscillometric method. Routine blood tests, including fasting plasma glucose, total cholesterol, high-density lipoprotein cholesterol (HDL-C), low-density lipoprotein cholesterol (LDL-C), serum uric acid (SUA) and serum triglycerides were measured by an Olympus AU640 auto analyzer (Olympus, Kobe, Japan). All blood samples were collected after an overnight 12-h fast at the same blood drawing station.

### The 3000-m run test

The participants ran a 3-km distance on a flat playground at the Hualien Military Physical Training and Testing Center without bearing a load. The run test was performed outdoor at 16:00 PM after at least a 1-h rest. The weather for the run test was restricted to the condition when there was no heavy raining and the heat stroke risk coefficient, defined as the outdoor temperature on the Celsius scale × relative humidity (%) × 0.1, was < 40^20^.

### Cardiometabolic risk markers for LVM

Based on the latest criteria of International Diabetes Federation for Chinese^21^, metabolic syndrome was defined as having three or more clinical features: (1) central obesity: WC ≥ 90 cm for men, and ≥ 80 cm for women; (2) fasting plasma glucose ≥ 100 mg/dL, or with anti-diabetic medications; (3) serum triglycerides ≥ 150 mg/dL, or with lipid-lowering medications; (4) HDL-C < 40 mg/dL for men, and < 50 mg/dL for women; (5) systolic BP ≥ 130 mmHg and/or diastolic BP ≥ 85 mmHg, or with antihypertensive medications. SUA has been associated with levels of oxidative stress and systemic inflammation in the body^22,23^. Hyperuricemia was defined as SUA ≥ 7.0 mg/dL for men and ≥ 6.0 mg/dL for women.

### ECG and echocardiographic measurements

Twelve-lead surface ECGs were obtained from CARDIOVIT MS-2015 machine (the Schiller AG, Baar, Switzerland) until a high-quality output image available. The ECG reports were reviewed and approved by an experienced cardiologist. The Sokolow-Lyon voltage criterion for LVH was defined as if the maximum of (S-V1 or V2 + R-V5 or V6) ≥ 35 mm^24^, and the Cornell voltage criterion for Asian young men was defined as if (R-aVL + S-V3) ≥ 18 mm^25^.

The transthoracic echocardiography (iE33; Philips Medical Systems, Andover, MA, USA) with a 1–5 MHz transducer, was reviewed by the same cardiologist at the Hualien-Armed Forces General Hospital. Based on the recommendations of the American Society of Echocardiography^26^, LVM was calculated at end diastole using the corrected formula proposed by Devereux et al.^27^: 0.8 × {1.04 × [(LV internal diameter (LVIDd) + posterior wall thickness + interventricular septal thickness]^3 ^− LVIDd^3^} + 0.6. Echocardiographic LVH was defined as the highest 5% of the LVM indexed for the body height (m^2.7^)^28^, corresponding to 46.0 g/m^2.7^ (5.99%) for men and 38.0 g/m^2.7^ for women in this study^25,29^.

### Statistical analysis

Demographic, anthropometric, time for a 3000-m run (endurance exercise performance), cardiometabolic risk markers, ECG and echocardiography characteristics of the military male and female personnel were presented as numbers (%) for categorical variables and mean ± standard deviation for continuous variables, respectively. Univariate linear regression was used to determine sex-specific correlation of each cardiometabolic risk marker with LVM index. Multiple linear regression was used to determine the independent cardiometabolic risk markers of LVM index in men and women, respectively. Furthermore, multiple logistic regression was used to determine the odds ratio (OR) of cardiometabolic risk markers with echocardiographic LVH (in model 1 without adjustment for endurance exercise performance, and in model 2 with adjustment for endurance exercise performance, and in model 3 with further adjustment for body weight) and with ECG-based LVH (model 1 and model 2) in men. As the number of outcome of interests (LVH) was too small (N < 15) for women to be analyzed, the results were only provided in supplemental tables. A value of *P* < 0.05 was regarded significant. All analyses were performed using SPSS version 25.0 for Windows (IBM Corp., Armonk, NY, USA). This study has been reviewed and approved by the Institutional Review Board of the Human Research Ethics Committee (HREC) of the Mennonite Christian Hospital (No. 16-05-008) in Hualien City, Taiwan, and written informed consent was obtained from all participants. In addition, all methods were performed in accordance with the relevant guidelines and regulations.

### Ethics approval and consent to participate

The Institutional Review Board (IRB) of the Mennonite Christian Hospital (No. 16-05-008) in Hualien of Taiwan approved access to the data for the CHIEF study, and written informed consent was obtained from all participants.

## Results

### Clinical features and laboratory findings

The clinical characteristics of men and women are provided in Table 1. The age of men and women were 27.5 and 26.0 years, respectively. In general, men had a greater level of BMI, WC, BP, fasting plasma glucose, total cholesterol, serum triglycerides, SUA, exercise performance (shorter time for a run test) and LVM, and a higher prevalence of active cigarette smoking, alcohol intake, metabolic syndrome and ECG-based LVH, compared to women. In contrast, men had a lower level of HDL-C level than women.

**Table 1:** Baseline Characteristics of the Military Men and Women (N = 2272.

	Men (N = 2019)	Women (N = 253)
Age, years	27.53 ± 5.93	26.02 ± 4.95
Smoking, active (%)	892 (44.18)	56 (22.13)
Alcohol intake, active (%)	854 (42.30)	44 (17.39)
3000-m running time, second	873.15 ± 93.58	1035.98 ± 117.68
Body mass index, kg/m^2^	24.88 ± 3.72	22.65 ± 2.89
Waist circumference, cm	83.56 ± 9.63	74.50 ± 7.77
Systolic blood pressure, mmHg	118.60 ± 13.14	106.79 ± 10.66
Diastolic blood pressure, mmHg	70.12 ± 10.28	64.72 ± 8.08
Total cholesterol, mg/dl	174.22 ± 34.09	168.55 ± 28.92
High-density lipoprotein, mg/dl	49.36 ± 10.44	60.70 ± 12.82
Serum triglycerides, mg/dl	108.29 ± 82.20	66.09 ± 29.35
Fasting plasma glucose, mg/dl	93.58 ± 11.61	90.37 ± 7.50
Serum uric acid, mg/dl	6.74 ± 1.35	4.75 ± 0.93
Metabolic syndrome (%)	255 (12.63)	8 (3.16)
LVM, gram	148.34 ± 32.01	103.34 ± 18.94
**ECG voltage criteria**		
Sokolow-Lyon based LVH (%)	1039 (51.46)	14 (5.53)
Cornell based LVH (%)	199 (9.86)	3 (1.19)
**Echocardiographic criteria**		
LVM index ≥ 46 g/m^2.7^ for men (%), or ≥ 38 g/m^2.7^ for women (%)	121 (5.99)	11 (4.30)

*ECG* Electrocardiography, *LVH* Left ventricular hypertrophy, *LVM* Left ventricular mass.

### The correlations of cardiometabolic risk markers with LVM index

The results of univariate and multiple linear regressions of cardiometabolic risk markers with LVM index in men and women are shown in Table 2. For men, in the univariate analysis, each cardiometabolic risk marker, except the endurance exercise performance, was significantly correlated with LVM index, whereas in the multiple linear regression, only age, systolic BP, time for a 3000-m run, serum triglycerides, SUA and WC were correlated with LVM index (β = 0.07, 0.10, − 0.01, 0.01, 0.24 and 0.24, respectively; all *p*-values < 0.05). For women, systolic BP, HDL-C and WC were correlated with LVM index in the univariate analysis (β = 0.07, − 0.05 and 0.32, respectively; all *p*-values < 0.05), whereas the correlation was merely significant for WC in the multivariable model (β = 0.20; *p* < 0.01). If body weight was further included in the multiple linear regression analysis, the association for WC was reduced more in women (β = 0.06, *p* = 0.36) than in men (β = 0.15, *p* < 0.01), indicating the effect of body weight higher than WC for LVM in women (R^2^ for the correlation between body weight and WC: 0.82 and 0.61 for men and women, respectively in supplemental Fig. 1).

**Table 2:** Sex-Specific Correlations between Cardiometabolic Risk Factors and LVMI.

	LVM/height^2.7^ (Men)							LVM/height^2.7^ (Women)						
	Univariate			Multivariate		Multivariate		Univariate			Multivariate		Multivariate	
	R	β	*p*	β	p	β	p	R	β	p	β	p	β	p
Age	0.18	0.23	< 0.01	0.07	< 0.01	0.08	< 0.01	0.002	− 0.002	0.97	− 0.05	0.43	− 0.05	0.45
3000-m running time	0.04	0.003	0.07	− 0.01	< 0.01	− 0.01	< 0.01	0.03	− 0.001	0.67	− 0.003	0.26	− 0.004	0.11
Systolic BP	0.33	0.18	< 0.01	0.10	< 0.01	0.10	< 0.01	0.14	0.07	0.02	0.05	0.20	0.04	0.28
Diastolic BP	0.25	0.18	< 0.01	0.01	0.50	0.01	0.49	0.05	0.03	0.39	− 0.02	0.62	− 0.02	0.68
Total cholesterol	0.12	0.03	< 0.01	− 0.01	0.29	− 0.004	0.42	0.03	0.006	0.60	0.01	0.35	0.02	0.22
HDL-C	0.15	− 0.11	< 0.01	− 0.01	0.51	− 0.01	0.47	0.13	− 0.05	0.04	− 0.05	0.09	− 0.05	0.07
Serum triglycerides	0.20	0.02	< 0.01	0.01	0.01	0.01	0.01	0.10	0.02	0.11	− 0.003	0.79	− 0.002	0.86
Fasting glucose	0.11	0.07	< 0.01	− 0.004	0.73	− 0.005	0.71	0.07	− 0.05	0.29	− 0.07	0.10	− 0.07	0.09
Serum uric acid	0.19	1.01	< 0.01	0.24	0.04	0.22	0.06	0.10	0.52	0.13	0.009	0.97	0.06	0.87
Waist circumference	0.42	0.32	< 0.01	0.24	< 0.01	0.15	< 0.01	0.32	0.21	< 0.01	0.20	< 0.01	0.06	0.36
Body weight	0.41	0.242	< 0.01			0.08	< 0.01	0.34	0.21	< 0.01			0.17	< 0.01

Multiple linear regression analysis was used to determine the association of LVM index with age, 3000-m running time, systolic BP, diastolic BP, total cholesterol, HDL-C, serum triglycerides, fasting glucose, serum uric acid, waist circumference, cigarette smoking and alcohol intake status, with and without body weight.

*BP* Blood pressure, *HDL-C* High-density lipoprotein cholesterol, *LVM* Left ventricular mass.

### Cardiometabolic risk markers for echocardiographic LVH in men

Table 3 reveals the results of multiple logistic regression analysis for the risk markers of echocardiographic LVH in men. Further adjustment for endurance exercise performance in model 2 did not alter the main results from model 1. Prehypertension, hypertriglyceridemia, hyperuricemia, abdominal obesity and metabolic syndrome were the independent risk markers of echocardiographic LVH in men in model 2 [ORs: 4.15 (95% confidence intervals (CI): 2.83–6.08), 1.78 (95% CI: 1.18–2.70), 2.28 (95% CI: 1.56–3.34), 3.38 (95% CI: 2.28–5.02) and 2.96 (95% CI: 1.94–4.52), respectively]. In model 3, with further adjustment for body weight, prehypertension and hyperuricemia remained the independent risk markers of echocardiographic LVH in men [ORs: 2.83 (95% CI: 1.89–4.23) and 1.67 (95% CI: 1.12–2.48), respectively], and there was a marginal association for metabolic syndrome [OR: 1.58 (95% CI: 0.99–2.50)]. However, the association for abdominal obesity was reduced and became insignificant (OR: 1.22, *p* = 0.46), probably due to a high correlation between body weight and WC. The results for women were provided in supplemental Table 1, which shows no significant associations for all cardiometabolic risk markers.

**Table 3:** Associations between Cardiometabolic Risk Factors and Echocardiographic Left Ventricular Hypertrophy in Men.

	LVM/height^2.7^ ≥ 46 g/m^2.7^					
	Model 1		Model 2		Model 3	
	OR (95% CI)	*p*-value	OR (95% CI)	*p*-value	OR (95% CI)	*p*-value
BP ≥ 130/85 mmHg	4.24 (2.90–6.21)	< 0.01	4.15 (2.83–6.08)	< 0.01	2.83 (1.89–4.23)	< 0.01
Total cholesterol ≥ 200 mg/dl	1.10 (0.71–1.69)	0.67	1.10 (0.71–1.70)	0.67	1.00 (0.64–1.55)	0.98
HDL-C < 40 mg/dl	1.42 (0.92–2.19)	0.11	1.36 (0.88–2.10)	0.16	0.96 (0.61–1.52)	0.86
Serum triglycerides ≥ 150 mg/dl	1.82 (1.20–2.75)	< 0.01	1.78 (1.18–2.70)	< 0.01	1.38 (0.90–2.11)	0.14
Fasting glucose ≥ 100 mg/dl	1.13 (0.73–1.76)	0.58	1.13 (0.73–1.76)	0.58	0.90 (0.57–1.43)	0.66
Serum uric acid ≥ 7.0 mg/dl	2.32 (1.59–3.40)	< 0.01	2.28 (1.56–3.34)	< 0.01	1.67 (1.12–2.48)	0.01
Waist circumference ≥ 90 mg/dl	3.53 (2.39–5.22)	< 0.01	3.38 (2.28–5.02)	< 0.01	1.22 (0.71–2.10)	0.46
Metabolic syndrome	3.11 (2.04–4.73)	< 0.01	2.96 (1.94–4.52)	< 0.01	1.58 (0.99–2.50)	0.05

Multiple logistic regressions were used to determine the association of cardiometabolic risk factors with echocardiographic left ventricular hypertrophy.

Model 1 adjusted for age, BP ≥ 130/85 mmHg, total cholesterol ≥ 200 mg/dl, HDL-C < 40 mg/dl, serum triglycerides ≥ 150 mg/dl, fasting glucose ≥ 100 mg/dl, serum uric acid ≥ 7.0 mg/dl, waist circumference ≥ 90 mg/dl, metabolic syndrome status, cigarette smoking status and alcohol intake status.

Model 2 adjusted for the covariates in model 1 and 3000-m running time.

Model 3 adjusted for the covariates in model 2 and body weight.

*BP* Blood pressure, *HDL-C* High-density lipoprotein cholesterol, *LVM* Left ventricular mass.

### Cardiometabolic risk markers for ECG-LVH in men

The results of multiple logistic regression analysis for the cardiometabolic risk markers of ECG-LVH in men are shown in Table 4. Additional adjustment for endurance exercise performance in model 2 yielded similar results compared to model 1. In model 2, prehypertension was the only independent risk marker of Sokolow-Lyon based ECG-LVH [ORs: 1.28 (95% CI: 1.03–1.60)]. However, abdominal obesity and metabolic syndrome uncovered an inverse association with Sokolow-Lyon based ECG-LVH [ORs: 0.54 (95% CI: 0.43–0.66) and 0.74 (95% CI: 0.56–0.97), respectively]. Prehypertension and hypertriglyceridemia were independent risk markers of Cornell based ECG-LVH [ORs: 1.65 (95% CI: 1.19–2.29) and 1.44 (95% CI: 1.01–2.04), respectively], whereas the associations of abdominal obesity and metabolic syndrome with Cornell based ECG-LVH were null. The results for women were demonstrated in supplemental Table 2, which revealed prehypertension to be the independent risk marker of both Sokolow-Lyon and Cornell based ECG-LVH, and metabolic syndrome to be another risk marker of Cornell based ECG-LVH.

**Table 4:** Associations between Cardiometabolic Risk Factors and Electrocardiographic Left Ventricular Hypertrophy in Men.

	Sokolow-Lyon based ECG-LVH				Cornell based ECG-LVH			
	Model 1		Model 2		Model 1		Model 2	
	OR (95% CI)	*p*-value	OR (95% CI)	*p*-value	OR (95% CI)	*p*-value	OR (95% CI)	*p*-value
BP ≥ 130/85 mmHg	1.26 (1.01–1.57)	0.04	1.28 (1.03–1.60)	0.03	1.66 (1.19–2.30)	< 0.01	1.65 (1.19–2.29)	< 0.01
Total cholesterol ≥ 200 mg/dl	0.87 (0.70–1.10)	0.24	0.87 (0.70–1.10)	0.24	0.95 (0.65–1.37)	0.76	0.95 (0.65–1.37)	0.76
HDL-C < 40 mg/dl	0.82 (0.65–1.03)	0.09	0.84 (0.67–1.06)	0.13	0.91 (0.62–1.33)	0.62	0.90 (0.61–1.32)	0.58
Serum triglycerides ≥ 150 mg/dl	1.00 (0.79–1.27)	0.98	1.02 (0.80–1.29)	0.89	1.44 (1.01–2.05)	0.04	1.44 (1.01–2.04)	0.04
Fasting glucose ≥ 100 mg/dl	0.83 (0.66–1.05)	0.12	0.84 (0.67–1.06)	0.13	0.89 (0.61–1.31)	0.55	0.89 (0.61–1.31)	0.55
Serum uric acid ≥ 7.0 mg/dl	0.83 (0.69–1.00)	0.05	0.84 (0.70–1.01)	0.07	1.03 (0.76–1.39)	0.87	1.02 (0.75–1.38)	0.90
Waist circumference ≥ 90 mg/dl	0.52 (0.43–0.65)	< 0.01	0.54 (0.43–0.66)	< 0.01	1.26 (0.91–1.74)	0.16	1.25 (0.90–1.73)	0.18
Metabolic syndrome	0.71 (0.54–0.94)	0.01	0.74 (0.56–0.97)	0.03	1.29 (0.86–1.95)	0.22	1.28 (0.85–1.93)	0.24

Multiple logistic regressions were used to determine the association of cardiometabolic risk factors with electrocardiographic LVH.

Model 1 adjusted for age, BP ≥ 130/85 mmHg, total cholesterol ≥ 200 mg/dl, HDL-C < 40 mg/dl, serum triglycerides ≥ 150 mg/dl, fasting glucose ≥ 100 mg/dl, serum uric acid ≥ 7.0 mg/dl, waist circumference ≥ 90 mg/dl, metabolic syndrome status, cigarette smoking status and alcohol intake status.

Model 2 adjusted for the covariates in model 1 and 3000-m running time.

*BP* Blood pressure, *HDL-C* High-density lipoprotein cholesterol, *LVH* Left ventricular hypertrophy.

## Discussion

The principal findings in the present study were that in physically active Asian young adults, the associations between cardiometabolic risk markers and LVM index may vary by sex. Greater age, systolic BP, serum triglycerides, SUA, body weight and WC, and superior endurance exercise performance were correlated with greater LVM index in men, whereas only greater WC or body weight was correlated with greater LVM index in women. In addition, in our male subjects, prehypertension, hypertriglyceridemia, hyperuricemia, abdominal obesity and metabolic syndrome were the independent risk markers of echocardioghraphic LVH. Prehypertension was the independent risk marker of both Sokolow-Lyon and Cornell based ECG -LVH. Hypertriglyceridemia was found as another risk marker of Cornell based LVH; however abdominal obesity and metabolic syndrome had a lower risk of Sokolow-Lyon based ECG-LVH.

Most prior studies were designed to investigate the association between cardio-metabolic risk markers and LVM index in hypertensive or diabetic individuals, who were at middle or old ages^2,30^, and the physical activity or fitness was rarely taken into account^6^. The independent cardiometabolic risk markers of LVM may vary according to the subjects selected. In hypertensive patients, age, systolic BP, fasting glucose, serum triglycerides and SUA were correlated with LVM^2,6,8^; however, the relationship for serum triglycerides might be only present in men, and the predictor of LVM in the overweight or obese was only systolic BP^31^. In contrast, in type 2 diabetic patients, Jørgensen et al. reported a correlation of systolic BP and HDL-C with LVM^30^, whereas there were no associations for hemoglobin A1c. With regard to the impact of physical activity, Joseph et al. showed the association between physical activity and LVM merely in the normotensive individuals^6^. In this present study, we further found a sex difference that the association for endurance exercise performance, an estimate for physical fitness, was present in men but not in women free of hypertension. Moreover, for physically active premenopausal women, we revealed that body weight or WC rather than systolic BP was the only independent metabolic risk marker for greater LVM.

Hypertension, abdominal obesity and metabolic syndrome have been revealed as the risk biomarkers of Cornell based ECG-LVH in the general population^32^. Similarly, the present study revealed that prehypertension was the metabolic risk marker of Sokolow-Lyon and Cornell based ECG-LVH in both physically active young men and women. Hypertriglycerdemia and metabolic syndrome were the independent risk biomarkers of Cornell based ECG-LVH in physically active men and women, respectively. On the contrary, our prior and present works revealed an inverse association with Sokolow-Lyon based ECG-LVH for men^10^. As compared to men, premenopausal women were thinner, and affected more by the breast than the chest wall thickness, which might result in the sex differences. In addition, the Cornell based ECG-LVH, unlike the Sokolow-Lyon based ECG-LVH, used the limb lead aVL voltage as a part of the criterion, and might reduce the effect of greater chest wall thickness in obesity on diminishing the electrical signals, which has shown a higher accuracy to echocardiographic LVH for both young men and women in prior studies^10^. Furthermore, using the Cornell based ECG-LVH was superior to using the Sokolow-Lyon based ECG-LVH to predict incident CVD, which has been shown in prior studies as well^33,34^. Accordingly, using the Cornell based ECG criterion for LVH is suggestive for identifying those who were at greater cardiometabolic risk from the physically young adults in the present study.

### Study strengths and limitations

The major strength of the CHIEF heart study was that the study subjects were included from the military in Taiwan, where the training was standardized. As the military base is a closed system, the environment and daily life for the study subjects are similar, which could reduce the unmeasured confounders. By contrast, there had some limitations in the present study. First, the sample size of women had an insufficient power for the multiple logistic regression analysis. Second, since this was a cross-sectional study, the temporal changes in cardiac structure could not be assessed.

## Conclusion

The study suggests that in physically active young adults, the associations of cardiometabolic risk markers with LVM and ECG-LVH may vary by sex. In men, there was an association of endurance exercise performance with LVM, however the associations of cardiometabolic risk markers with LVM were not much modified by the exercise performance. In addition, greater body weight or WC, indicating obesity, was a powerful risk marker of greater LVM in both men and women. Thus, weight reduction is suggested to reduce LVM and for a prevention of incident CVD. On the other respect, prehypertension was the risk marker of Cornell and Sokolow-Lyon based ECG-LVH for both men and women, while not the risk marker of echocardiographic LVH for women. This sex difference reflects that a mildly elevated BP level may increase the power of cardiac electricity prior to the structural change of LVH, particularly for the physically young women. Further studies are required to clarify this finding.

## Supplementary Information

Supplementary Information 1.

Supplementary Information 2.

## Data Availability

The datasets generated and/or analysed during the current study are not publicly available due to materials obtained from the military in Taiwan, which were confidential, but are available from the corresponding author on reasonable request.

## Abbreviations

CHIEF: Cardiorespiratory fitness and health in eastern armed forces study
LVH: Left ventricular hypertrophy
LVM: Left ventricular mass
