# Supplementary figures and images for: Sex-specific cardiometabolic risk markers of left ventricular mass in physically active young adults: the CHIEF heart study
Source: Sci Rep. 2022 Jul 7;12:11536. doi: 10.1038/s41598-022-15818-y (PMC9263143; doi:10.1038/s41598-022-15818-y)

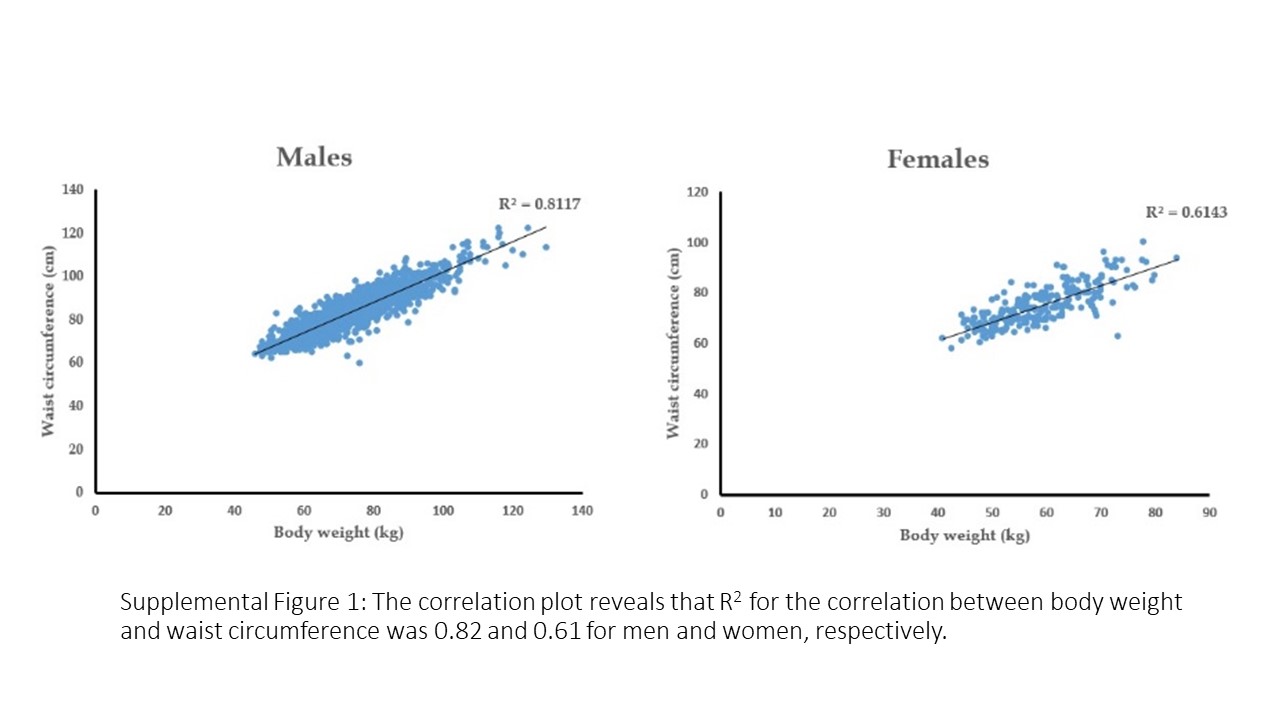

Supplement: Supplementary file 1 — Supplementary Information 1. [file 41598_2022_15818_MOESM1_ESM.jpg]
