# Supplementary material for: Sex-specific cardiometabolic risk markers of left ventricular mass in physically active young adults: the CHIEF heart study
Source: Sci Rep. 2022 Jul 7;12:11536. doi: 10.1038/s41598-022-15818-y (PMC9263143; doi:10.1038/s41598-022-15818-y)
Supplement: Supplementary file 2 — Supplementary Information 2. [file 41598_2022_15818_MOESM2_ESM.docx]

**Supplemental Table 1.** Associations between Cardiometabolic Risk Markers and Echocardiographic Left Ventricular Hypertrophy in Women

|  | LVM/height^2.7^ ≥46 g/m^2.7^ | | | | | | | |
| --- | --- | --- | --- | --- | --- | --- | --- | --- |
|  | Model 1 | |  | Model 2 | |  | Model 3 | |
|  | OR (95% CI) | p-value |  | OR (95% CI) | p-value |  | OR (95% CI) | p-value |
| BP ≥130/85 mmHg | 3.12 (0.59 – 16.58) | 0.18 |  | 3.05 (0.57 – 16.23) | 0.19 |  | 2.43 (0.44 – 13.57) | 0.31 |
| Total cholesterol ≥200 mg/dl | 1.31 (0.41 – 4.15) | 0.64 |  | 1.38 (0.43 – 4.39) | 0.58 |  | 1.44 (0.45 – 4.66) | 0.54 |
| HDL-C <40 mg/dl | 0.75 (0.24 – 2.33) | 0.61 |  | 0.69 (0.21 – 2.20) | 0.52 |  | 0.63 (0.20 – 2.02) | 0.43 |
| Serum triglycerides ≥150 mg/dl | 0.00 (0.00 - ) | 0.99 |  | 0.00 (0.00 - ) | 0.99 |  | 0.00 (0.00 - ) | 0.99 |
| Fasting glucose ≥100 mg/dl | 2.30 (0.70 – 7.57) | 0.17 |  | 2.32 (0.70 – 7.67) | 0.16 |  | 2.15 (0.64 – 7.27) | 0.21 |
| Serum uric acid ≥7.0 mg/dl | 0.74 (0.16 – 3.35) | 0.69 |  | 0.66 (0.14 – 3.17) | 0.60 |  | 0.61 (0.13 – 2.87) | 0.53 |
| Waist circumference ≥90 mg/dl | 2.07 (0.85- 5.05) | 0.10 |  | 2.00 (0.81 – 4.93) | 0.13 |  | 1.10 (0.33 – 3.70) | 0.87 |
| Metabolic syndrome | 3.55 (0.65 – 19.36) | 0.14 |  | 3.39 (0.62 – 18.65) | 0.16 |  | 2.26 (0.39 – 13.04) | 0.36 |

Multiple logistic regressions were used to determine the association of cardiometabolic risk factors with echocardiographic left ventricular hypertrophy.

Model 1 adjusted for age, BP ≥130/85 mmHg, total cholesterol ≥200 mg/dl, HDL-C <50 mg/dl, serum triglycerides ≥150 mg/dl, fasting glucose ≥100 mg/dl, serum uric acid ≥6.0 mg/dl, waist circumference ≥80 mg/dl, metabolic syndrome, smoking and alcohol intake

Model 2 adjusted for the covariates in model 1 and 3000-m running time

Model 3 adjusted for the covariates in model 2 and body weight

Abbreviations: BP, blood pressure; HDL-C, high-density lipoprotein cholesterol, LVM, left ventricular mass

**Supplemental Table 2.** Associations between Cardiometabolic Risk Markers and Electrocardiographic Left Ventricular Hypertrophy in Women

|  | Sokolow-Lyon based LVH | | | | |  | Cornell based LVH | | | | |
| --- | --- | --- | --- | --- | --- | --- | --- | --- | --- | --- | --- |
|  | Model 1 | |  | Model 2 | |  | Model 1 | |  | Model 2 | |
|  | OR (95% CI) | p-value |  | OR (95% CI) | p-value |  | OR (95% CI) | p-value |  | OR (95% CI) | p-value |
| BP ≥130/85 mmHg | 6.39 (1.13 – 35.99) | 0.03 |  | 6.82 (1.14 – 40.74) | 0.03 |  | 28.60 (1.49 – 550.11) | 0.02 |  | 30.35 (1.51 – 610.59) | 0.02 |
| Total cholesterol ≥200 mg/dl | 0.48 (0.06 – 3.93) | 0.49 |  | 0.41 (0.05 – 3.445) | 0.41 |  | 0.000 (0.000 - ) | 0.99 |  | 0.000 (0.000 - ) | 0.99 |
| HDL-C <50 mg/dl | 0.61 (0.13 – 2.86) | 0.52 |  | 0.69 (0.14 – 3.31) | 0.64 |  | 8.60 (0.72 – 103.27) | 0.09 |  | 8.34 (0.67 – 103.56) | 0.09 |
| Serum triglycerides ≥150 mg/dl | 0.000 (0.000 - ) | 0.99 |  | 0.000 (0.000 - ) | 0.99 |  | 0.00 (0.00 - ) | 0.99 |  | 0.00 (0.00 - ) | 0.99 |
| Fasting glucose ≥100 mg/dl | 0.84 (0.10 – 6.89) | 0.86 |  | 0.84 (0.10 – 6.95) | 0.87 |  | 0.00 (0.00 - ) | 0.99 |  | 0.00 (0.00 - ) | 0.99 |
| Serum uric acid ≥6.0 mg/dl | 0.61 (0.08 – 4.96) | 0.64 |  | 0.65 (0.08 – 5.36) | 0.69 |  | 3.59 (0.30 -42.60) | 0.31 |  | 3.01 (0.21 – 42.85) | 0.41 |
| Waist circumference ≥80 mg/dl | 0.92 (0.26 – 3.20) | 0.88 |  | 1.04 (0.29 – 3.69) | 0.95 |  | 6.92 (0.49 – 97.42) | 0.15 |  | 6.52 (0.46 – 91.92) | 0.16 |
| Metabolic syndrome | 2.36 (0.26 – 21.66) | 0.44 |  | 2.93 (0.31 – 27.79) | 0.35 |  | 14.04 (1.03 – 191.06) | 0.04 |  | 13.65 (1.00 – 186.91) | 0.05 |

Multiple logistic regressions were used to determine the association of cardiometabolic risk factors with electrocardiographic left ventricular hypertrophy.

Model 1 adjusted for age, BP ≥130/85 mmHg, total cholesterol ≥200 mg/dl, HDL-C <50 mg/dl, serum triglycerides ≥150 mg/dl, fasting glucose ≥100 mg/dl, serum uric acid ≥6.0 mg/dl, waist circumference ≥80 mg/dl, metabolic syndrome, smoking and alcohol intake

Model 2 adjusted for the covariates in model 1 and 3000-m running time

Abbreviations: BP, blood pressure; HDL-C, high-density lipoprotein cholesterol, LVM, left ventricular mass
